# A non-invasive olfactory bulb measure dissociates Parkinson’s patients from healthy controls and discloses disease duration

**DOI:** 10.1038/s41531-021-00220-8

**Published:** 2021-08-18

**Authors:** Behzad Iravani, Artin Arshamian, Martin Schaefer, Per Svenningsson, Johan N. Lundström

**Affiliations:** 1 Department of Clinical Neuroscience, Karolinska Institutet, Stockholm, Sweden; 2 Department of Psychology, Stockholm University, Stockholm, Sweden; 3 Monell Chemical Senses Center, Philadelphia, PA USA; 4 Department of Psychology, University of Pennsylvania, Philadelphia, PA USA; 5 Stockholm University Brain Imaging Centre, Stockholm University, Stockholm, Sweden

**Keywords:** Diagnostic markers, Parkinson's disease

## Abstract

Olfactory dysfunction is a prevalent non-motor symptom of Parkinson’s disease (PD). This dysfunction is a result of neurodegeneration within the olfactory bulb (OB), the first processing area of the central olfactory system, and commonly precedes the characteristic motor symptoms in PD by several years. Functional measurements of the OB could therefore potentially be used as an early biomarker for PD. Here, we used a non-invasive method, so-called electrobulbogram (EBG), to measure OB function in PD and age-matched healthy controls to assess whether EBG measures can dissociate PDs from controls. We estimated the spectrogram of the EBG signal during exposure to odor in PD (*n* = 20) and age-matched controls (*n* = 18) as well as identified differentiating patterns of odor-related synchronization in the gamma, beta, and theta frequency bands. Moreover, we assessed if these PD-EBG components could dissociate PD from control as well as their relationship with PD characteristics. We identified six EBG components during the initial and later stages of odor processing which dissociated PD from controls with 90% sensitivity and 100% specificity with links to PD characteristics. These PD-EBG components were related to medication, disease duration, and severity, as well as clinical odor identification performance. These findings support using EBG as a tool to experimentally assess PD interventions, potentially aid diagnosis, and the potential development of EBG into an early biomarker for PD.

## Introduction

Given the notion that the olfactory bulb (OB) is an indispensable olfactory processing node that is central for many olfactory tasks including, but not limited to, odor discrimination, concentration-invariant odor recognition, odor segmentation, and odor pattern recognition^1^, olfactory dysfunction is expected to follow impairment to its function. OB is further one of the first sites of insult in the central nervous system in Parkinson’s disease (PD). Under the current concept of PD’s pathology progression, Lewy bodies initiate in the OB and subsequently infiltrate to other neural systems, including olfactory and non-olfactory areas^2^. As a consequence, the olfactory deficit is a common early symptom in PD which often precedes the characterizing motor symptoms by several years^3,4^. Olfactory loss has therefore been proposed as a possible biomarker preceding PD clinical diagnosis^5^. Structural MRI approaches have reported that PD is associated with reduced OB volume^6^ and fractional anisotropy, measured by diffusion tensor imaging (DTI)^7^. However, because the OBs are situated in an area susceptible to MR artifacts, the variations between the DTI series are higher than for other brain regions. Moreover, destruction of a large portion of the OB is required before a reduction in olfactory behavioral performance can be detected^8^, and performance on behavioral tests is confounded by cognitive abilities^9^. Hence, a direct measure of OB neural responses to odors would be an ideal candidate for a potential early indication of PD that is not dependent on cognitive abilities.

Odor processing has repeatedly been used to dissociate PD patients from healthy controls based on cerebral responses using either EEG^10–12^ or fMRI^13,14^ measures where several promising results have emerged. All studies to date, however, have obtained results that are more likely linked to either cognitive aspects of the odor percept or indirect measures of OB responses, such as differences in early ERP responses^10^. This lack of measures from the OB can be explained by the inherent limitations of the various methods used to date that render them unable to assess functional non-invasive measures from the human OB. However, it was recently demonstrated that a reliable odor-related OB signal can be assessed by means of a so-called electrobulbogram (EBG), a non-invasive method that can reliably measure functional OB processing of odors from micro-amplified electrodes on the forehead^15^. The odor-related EBG response in healthy human participants is defined as an early signal in the gamma band which is thought to reflect mostly within-OB processing^16^. Interestingly, in vivo recordings from the OB in animal PD models have demonstrated that partial depletion of dopaminergic neurons in the substantia nigra decreases the odor-induced activity in both the gamma and beta bands within the OB^17^. Taken together, these findings suggest that odor-evoked oscillatory processing within the OB could be an early and sensitive indication of PD.

Previously, the EBG method has been used to assess valence processing^18^ as well as the communication between OB and olfactory cortex in humans^19^ where it was demonstrated that OB functions and communicates in multiple frequency bands. Particularly, the afferent connection of OB was demonstrated to operate in the gamma/beta bands whereas the efferent connection of OB in the theta/delta bands^19^. As mentioned earlier, the past rodent studies indicated that the gamma band oscillation in OB is linked to intra-bulb processes^20,21^ whereas the theta band is related to respiration^21^. Nevertheless, theta oscillations in humans have been indicated that have more relevance in odor processing especially in higher-order olfactory regions rather than mere respiration^22^. In the current study, we used this newly developed technique of non-invasive recording from the human OB (i.e., EBG) to assess whether odor-evoked oscillatory processing within the OB can differentiate diagnosed PD patients from healthy controls. We further determined the sensitivity and specificity of classification for EBG versus a standardized clinical olfactory test. Finally, we assessed whether these EBG responses were associated with standard measures of PD disease progression and severity.

## Results

Compared to Controls, PD patients demonstrated a clear olfactory impairment when assessed using a 16-item odor identification (odor ID) test^23^, *t*(36) = 7.70, *p* < 0.001, *CI* = [4.77, 8.18]. Participants’ OB responses were then assessed by exposing them to 1 s long stimuli consisting of either odor (3 iso-intense odors with diverse quality) or non-odorized air (to remove sniff only response) using 4 EBG electrodes attached on participants’ forehead. Initially, to assess potential differences in odor-dependent OB responses between PD and Control, the spectrogram of the EBG signal was estimated using a multi-taper convolution method, and, subsequently, sniff and evoked responses were removed. In addition, we assessed whether the differences found in the EBG spectrogram can be used to distinguish PDs from Controls and their relationship with PD parameters.

### EBG measure in PD and healthy Control

**Fig. 1:**
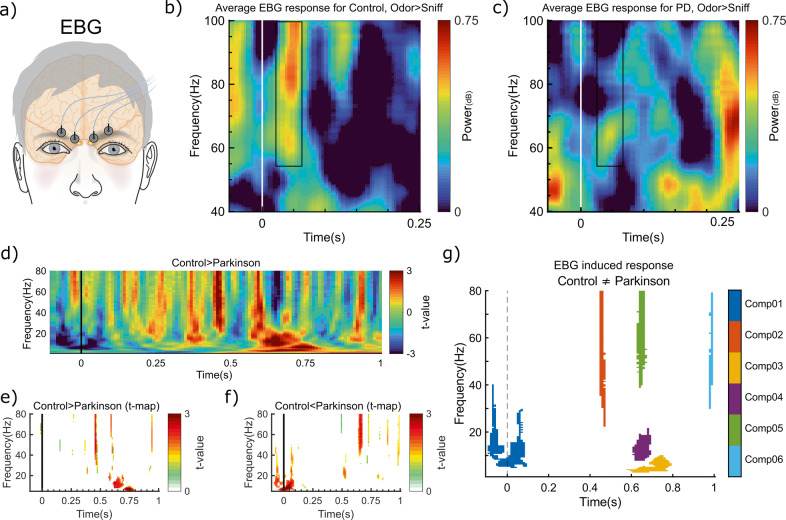
EBG measure can dissociate PD from control. **a** Placement of EBG electrodes on the forehead. **b** Odor-induced EBG response in the healthy controls replicates earlier studies. Gamma synchronization appears briefly after the odor onset (indicated by the horizontal white line at Time 0) for Controls. **c** No gamma synchronization was found for PDs in the same time period (EBG area of interest was marked with a black rectangle). Warmer colors indicate synchronization whereas cooler colors indicate desynchronization compared to Sniff (clean air). **d** T-map derived from 1000 Monte Carlo permutation tests. Early and late differences between Control and PD are statistically meaningful in theta, beta, and gamma band. **e** Threshold t-maps indicating areas with *p* < 0.05 where power is more for Control compared with PD. **f** Threshold t-maps indicating areas with *p* < 0.05 where power is less for Control compared with PD. Warmer colors in the t-maps represent higher *t*-values. **g** Clusters of significance (*p* < 0.05, cluster size > 100) differentiated EBG components that dissociate Control from PD. Specifically, we isolated six different components in the gamma, beta, and theta bands during the early and late time points. Each component is illustrated with a specific color and color labels can be found in the color bar on the right side of the panel.

First, we replicated the healthy EBG odor response in Controls using the same method as in Iravani et al.^15^. The location of EBG electrodes is illustrated in Fig. 1a. As predicted, we detected an early gamma synchronization after sniffing an odor in the EBG electrodes, Fig. 1b, a result similar to what has previously been described for the EBG response^15^. This validated that we could detect a robust OB signal in Controls in the present dataset. However, when we assessed the EBG spectrogram at the same time and frequency intervals in PD patients, no gamma synchronization was detected, Fig. 1c. Next, we assessed whether the EBG response to odors differed between PD and Control using a multi-taper sliding window to produce time-frequency maps of the EBG odor response. We found that the control group, when contrasted against the PD group and while controlling for a sniff and evoked responses, had greater odor-induced power in the theta, beta, and gamma band, Fig. 1d. To isolate specific areas that are significantly different between the two groups, we subsequently used non-parametric tests with 1000 permutations and found that the increases in power were significant for Control, compared to PD, in the gamma band around 460 ms after odor onset, *t*(37) = 3.28, *p* < 0.001, the beta band around 620 ms, *t*(37) = 2.7, *p* < 0.006, and finally in the theta band around 680 ms, *t*(37) = 3.87, *p* < 0.001, Fig. 1e. Comparably, we found a decrease in the power of gamma frequency around 660 ms, *t*(37) = 3.13, *p* < 0.002, as well as 980 ms, *t*(37) = 2.23, *p* < 0.012, and alpha/beta around odor onset, *t*(37) = 3.50, *p* < 0.02, in Control group compared with PD, Fig. 1f. We subsequently labeled these significant clusters (*p* < 0.05, cluster size > 100) based on the order of appearances as Component 1, Component 2, etc. A total of six components were identified, as illustrated in Fig. 1g. Here, Components 2, 3, and 4 had higher power in Controls whereas Components 1, 5, and 6 had lower power in Controls (i.e., higher power in PDs).

To assess the generalizability of our findings and to determine whether the differences in spectrogram between PD and Control are not dependent on a group level effect but also exist on the trial level, we performed intra-class correlation, namely ICC(2,k), to evaluate the level of agreement of each component across individuals in the two groups. We found medium to high ICC outcomes [0.46–0.78] for all detected components (Supplementary Fig. 1), thereby suggesting that these results are likely to replicate in an independent sample with similar individual characteristics.

### EBG measure versus odor ID in dissociating PD from healthy Control

We found six significant clusters in the time-frequency map of odor EBG responses that allowed us to differentiate between PD and Control. Next, we determined the sensitivity and specificity of the EBG results, as well as assessed how the EBG measures compared to a standard 16-item clinical cued odor ID test, using mixed-effect logistic regression models with the EBG components as independent variables.

**Fig. 2:**
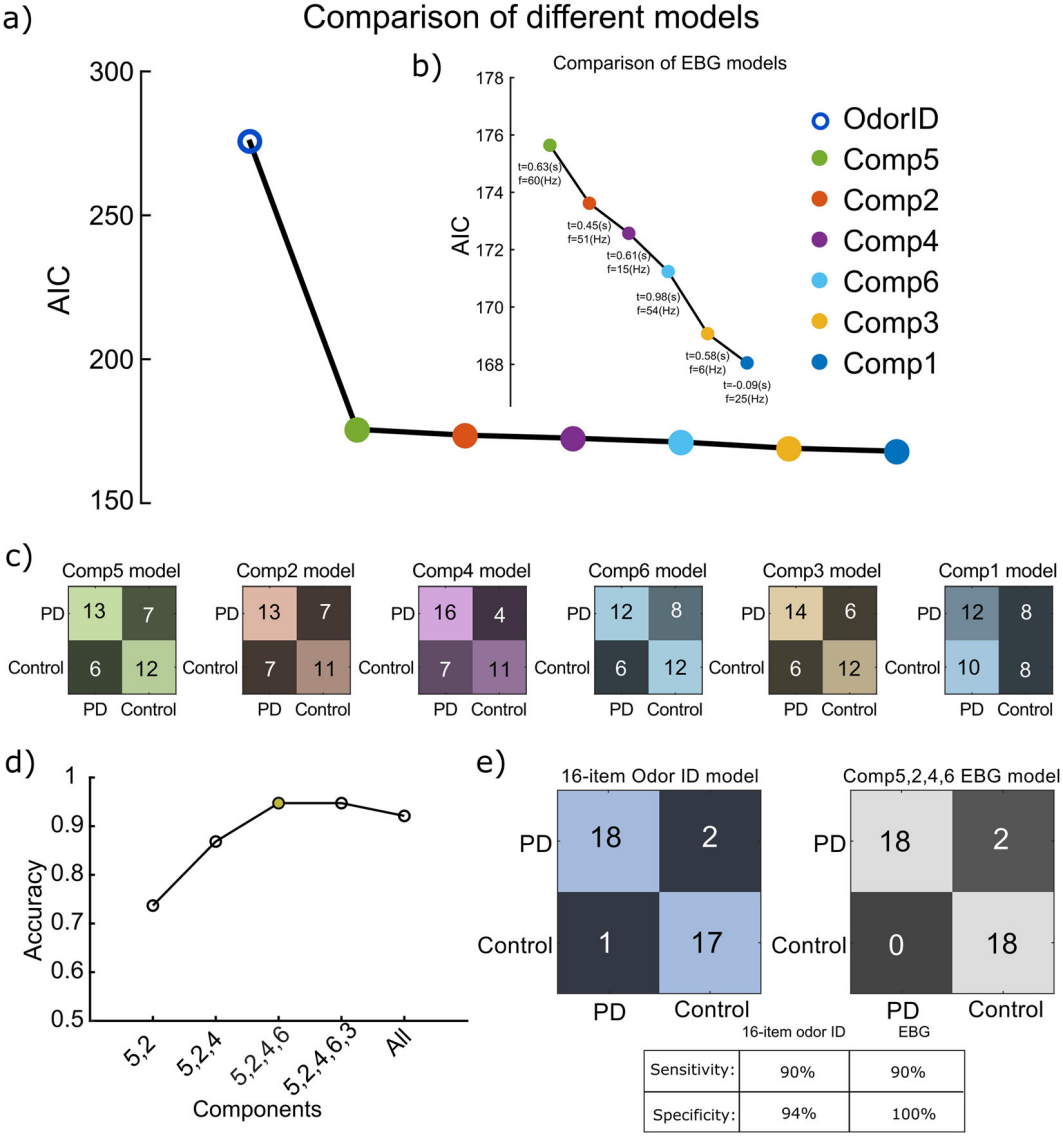
Comparison within EBG models as well as EBG and odor identification models. **a** Akaike information criteria (AIC) for each EBG component and 16-item odor ID. **b** Zoomed-in AIC values for the only EBG models. **c** Cross table of each component of the EBG models shows true/false positive/negative values. The colors of the EBG components in the panel (**a**), (**b**) and (**c**) correspond with Fig. 1g. **d** Accuracy of models with stepwise adding components shows maximum accuracy for the model includes components 5, 2, 4, and 6. Peak accuracy is marked with a filled yellow circle. **e** Crosstables of 16-item odor ID and multicomponent EBG model show comparable sensitivity and higher specificity, yet we found slightly more in-sample error compare with 16-item odor ID.

We first assessed the goodness of the fit for each EBG component by in-sample prediction error estimated by means of Akaike information criteria (AIC). We found that all EBG components’ AIC value was lower than the 16-item odor ID model. This means that the EBG models fit the obtained result better than the 16-item odor ID test, Fig. 2a. However, some EBG models worked better than others. For example, the model with Component 1 had the lowest AIC, 168.05, and outperformed the rest of the EBG models (Component 3 (AIC = 169.07), Component 6 (AIC = 171.24), Component 4 (AIC = 172.57), Component 2 (AIC = 173.62), and Component 5 (AIC = 175.64), Fig. 2b. However, these models are not nested, and assessing only in-sample error might not be sufficient. Therefore, we further assessed the sensitivity and specificity of each model. We did not achieve high sensitivity and specificity with models including only one EBG component, Fig. 2c. Therefore, we stepwise added components to the logistic regression and assessed prediction accuracy. We found a maximum accuracy of 94% with an AIC = 279.11 for a model including 4 components (Components 5, 4, 2, and 6), Fig. 2d. This model demonstrated a similar fit to the data as the 16-item odor model (AIC = 276.27). We then explored the sensitivity and specificity of the multicomponent EBG model. The odor ID model produced high values (sensitivity 90%; specificity 94%) whereas the multicomponent EBG resulted in 90% sensitivity and 100% specificity, Fig. 2e; this can be viewed as high values but nominally with more in-sample error compared with the 16-item odor ID model.

**Fig. 3:**
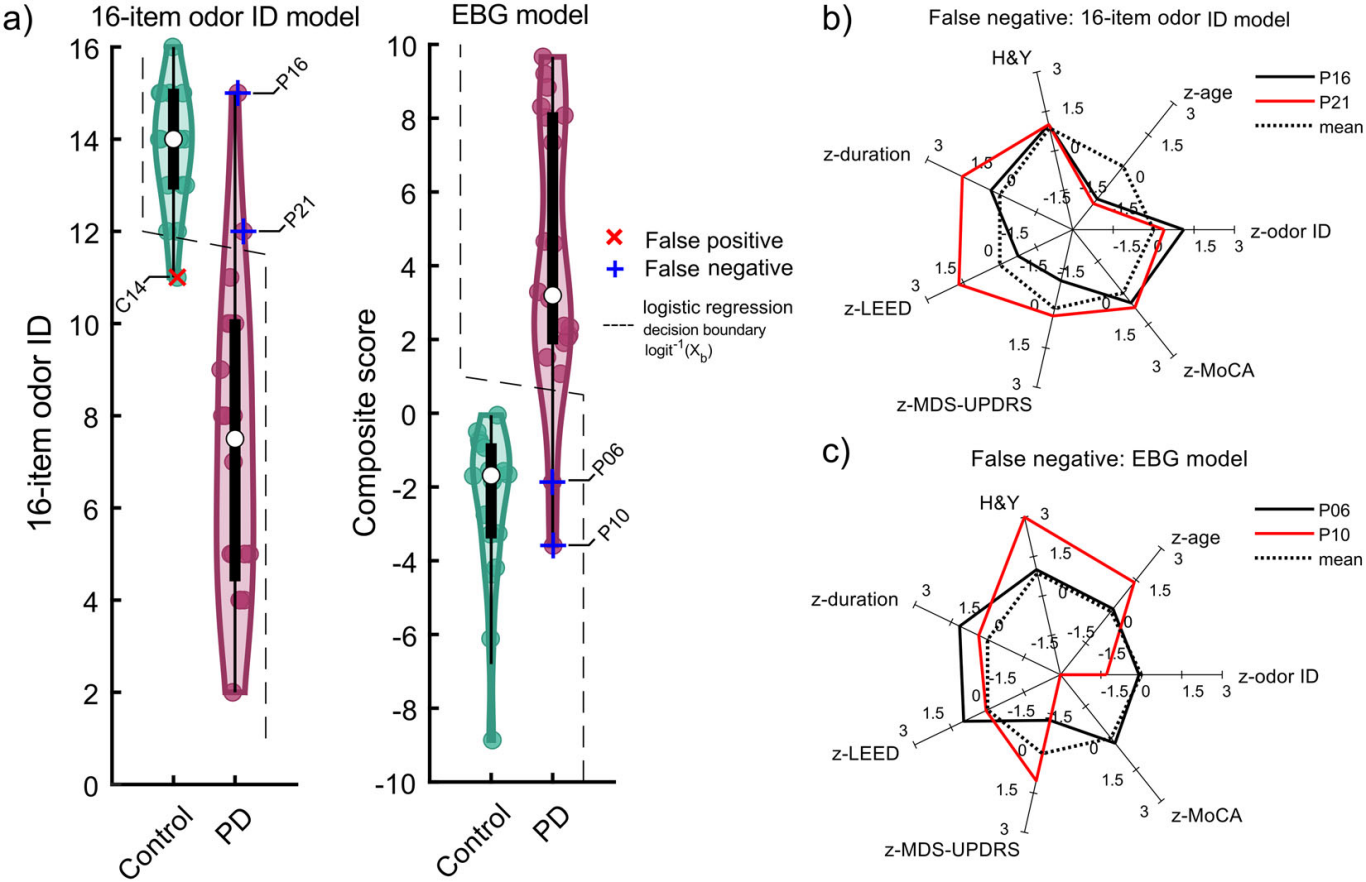
The logistic regression decision boundary. **a** Violin plot shows the distribution for 16-item odor ID responses and EBG responses. The scatter plot within the distribution shows individual responses. The black box shows the 25 and 75 percentiles in which the white dot shows the median. The whiskers show the maximum and minimum within 1.5 times of the interquartile range. The logistic decision boundary is shown with the dashed line. **b** Radar plot shows the demographic and PD parameters for the false negatives that occurred in the 16-item odor ID model. **c** Similarly, the radar plot shows the demographic and PD parameters for the false negatives that occurred in the EBG model. The dashed line in (**b**) and (**c**) shows the mean response. Note, all the false negatives had an available MDS-UPDRS score.

The cross table in Fig. 2e demonstrates that 1 individual was a false positive and 2 false negatives for the 16-item odor ID model whereas the EBG model produced 2 false-negative individuals. We subsequently assessed occurred errors in the models by plotting the decision boundary and identifying those individuals who were erroneously labeled. This analysis indicated that the two individuals who were incorrectly classified in the two models were not the same across models, Fig. 3a. Regarding the 16-item odor ID model, misclassifications are straightforward. Patients who did not develop hyposmia are here wrongly classified as control participants and the Control participant who was on the border of hyposmia was falsely classified as PD, Fig. 3b. The EBG model assigned all these individuals correctly to their respective groups. However, two other individuals were wrongly classified (false negatives) as healthy participants. One of the false negatives in the EBG model had more severe PD symptoms and cognitive deficits than the PD group as a whole, Fig. 3c.

### EBG components are linked to clinical PD measures

**Fig. 4:**
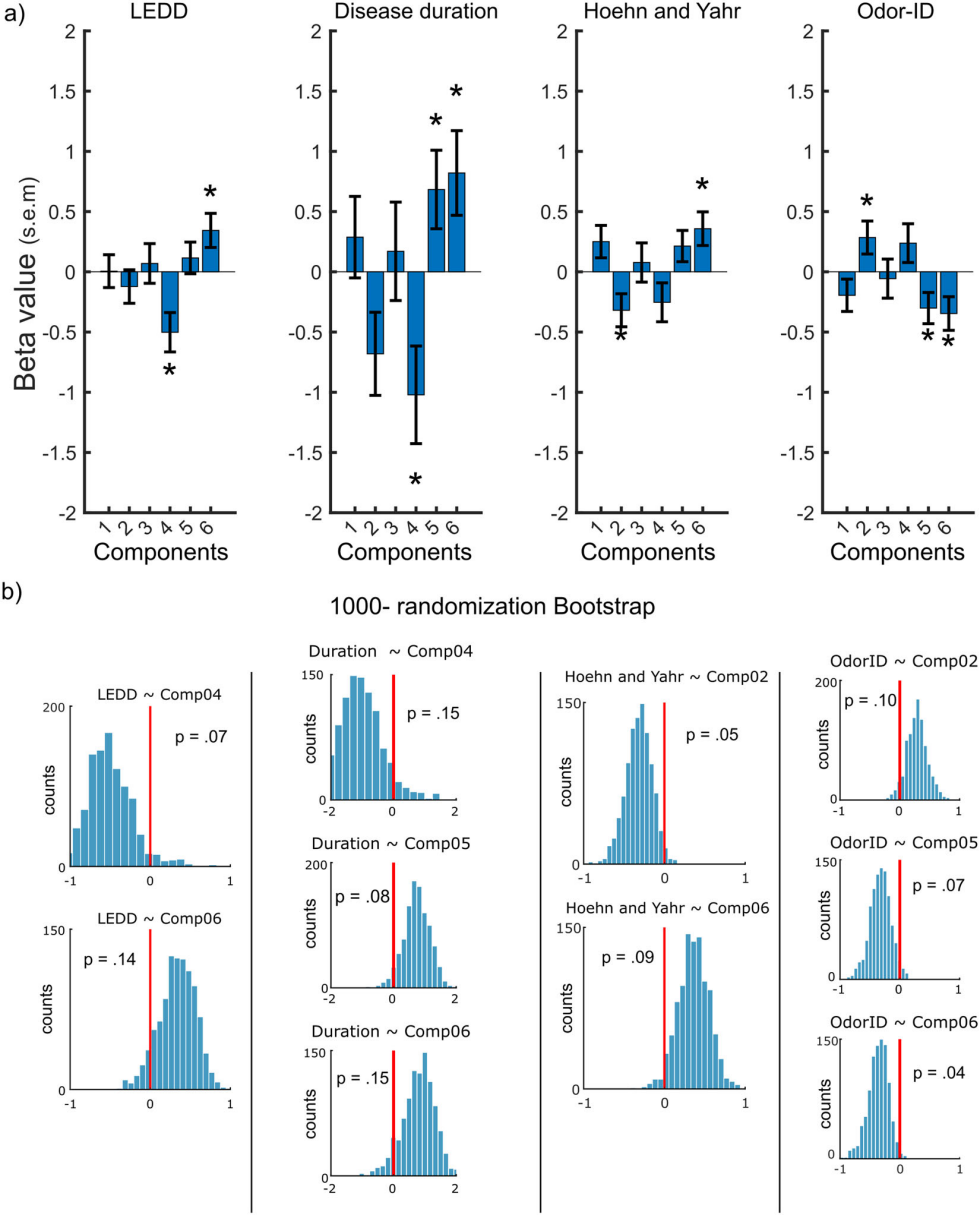
EBG components are associated with PD and olfactory performance. **a** Beta values for EBG components that associate with Levodopa equivalent daily dose (LEDD), disease duration, Hoehn & Yahr (H&Y) disease severity, and odor ID performance in PD individuals. Error bars show the standard error of the mean (s.e.m). Asterisks show significant components (*p* < 0.05). **b** Thousand-randomization bootstrap test was carried out for the significant components and exact *p*-values were subsequently estimated.

Having established that the EBG measure could distinguish PDs from controls, we next assessed whether the obtained results were linked to individual clinical PD disease parameters. To this end, we determined the potential relationships between the obtained EBG components and clinical measures using mixed-effect linear regression models. Specifically, we determined whether levels of the identified EBG components were associated with LEDD (i.e., medication dosage), disease duration, Hoehn and Yahr disease severity, and results on the 16-item ID test. We found that the values of both Component 4, *t*(31) = −3.07, *p* < 0.004, *CI* = [−0.83, −0.17], and Component 6, *t*(31) = 2.43, *p* < 0.021*, CI* = [0.055, 0.63], significantly predicted LEDD scores. Moreover, Component 4, *t*(31) = −2.52, *p* < 0.017, *CI* = [−1.85, −0.19], Component 5, *t*(31) = 2.10, *p* < 0.018, *CI* = [0.018, 1.35], and Component 6, *t*(31) = 2.34, *p* < 0.026, *CI* = [0.10, 1.54], were significantly related to disease duration. For PD H&Y disease severity, Component 2, *t*(31) = −2.35, *p* < 0.027, *CI* = [−0.60, −0.039], and Component 6, *t*(31) = 2.56, *p* < 0.015, *CI* = [0.073, 0.64], were significant. Finally, there were significant relationships between the values of Component 2, *t*(31) = 2.07, *p* < 0.046, *CI* = [0.005, 0.56], Component 5, *t*(31) = −2.33, *p* < 0.027, *CI* = [−0.56, −0.037], as well as Component 6, *t*(31) = −2.48*, p* < 0.018, *CI* = [−0.63, −0.062], and odor ID performance, Fig. 4a. Moreover, no correlation was found between the EBG components and age (all *p*-values above 0.18), but there was a significant negative correlation between MoCA and Component 3, *r* (16) = −0.60, *p* < 0.009, as well as Component 4, *r*(16) = −0.49, *p* < 0.04, in PDs.

Finally, to further validate associations between the EBG components and clinical PD parameters, we resampled data using 1000-randomization bootstrap and extracted *p*-values. However, only two of the associations survived the bootstrap test, namely the association between H&Y disease severity and Component 2 as well as the association between odor ID and Component 6, Fig. 4b. The remaining associations demonstrated reduced statistical significance with the largest *p*-value being *p* = 0.15.

## Discussion

Objective measurement of OB’s function has the potential to enable early diagnosis of PD, possibly several years before motor symptom onset. We previously found that EBG can reliably measure the function of OB in healthy participants^15^. Moreover, EBG was demonstrated to have high test-retest reliability (*r* = 0.81) similar to that of visual event-related potentials^15^. Here, we determined whether the EBG measure can be used as a PD detection tool by assessing whether the EBG measure alone is able to dissociate diagnosed PD patients from age-matched non-PD controls. Signals obtained using the EBG measure allowed us to identify OB response components, spread across frequencies and time intervals, that enabled us to discriminate between the PD and the control group. Individual EBG components were further demonstrated to be associated with clinical outcome parameters. Moreover, using logistic regression models, the EBG measure was able to dissociate PDs from Controls on the individual level to the same degree as a model based on odor ID performance. Albeit further development is needed before implementation as a clinical tool, these proof-of-concept results indicate that the EBG measure might be used as a potential early marker of PD.

We found different patterns of EBG signal’s power between Controls and PD patients in the gamma, beta, and theta bands during odor processing. Because the gamma band is mainly attributed to the within-OB processes^20,24^, the detected differences support the well-established neuronal loss-dependent impairment of OB processing in PD^8^. Moreover, activities in the beta/theta bands are likely generated by top-down projections^16^ which is further supported by their late latency in the present data. This result suggests an impairment in the bi-directional interaction between the OB and higher olfactory cortex regions (e.g., amygdala and the orbitofrontal cortex) that are involved in processing different aspects of odor perception and valence^25^. Notably, a behavioral study has found that odor valence perception is impaired in PD^26^, and in the results presented within this paper, the majority of identified components are relatively late in the neural processing cascade. This might be of special relevance given that previous olfactory event-related potential studies in PD patients have shown that the amplitude of the late components is lower compared to the healthy controls^10^, hence indicating that OB upstream communication is inhibited. The exact involvement of various brain areas in the obtained results should be assessed in future studies. However, it is worth noting that the sample size of this study is relatively small. Therefore, to discern the generalizability of our findings, we estimated the ICC which demonstrated medium to high agreement of Components across participants on the trial level. These ICC results suggest that it is likely that our findings can be replicated in a larger and independent sample.

The model including EBG components fitted the data with a slightly higher specificity than the 16-item odor ID model. Moreover, the false negatives occurring in the 16-item odor ID model were correctly assigned to the PD cohort in the EBG model. This result suggests that EBG, a measure that does not rely on cognitive and individualized judgment, can potentially be used to dissociate PDs from Controls. One of the early EBG components (Component 2) demonstrating the strongest correlation with PD disease severity appeared in the gamma band, a frequency attributed to within-OB processing. Hence, this potentially means that it is within-OB processing, or initial OB processing, that demonstrates the closest link to behavioral odor measures in PD. Moreover, no correlation was found between this component and cognitive measure, thereby suggesting that Component 2 is less dependent on the higher-order brain function. Previous studies suggest that olfactory loss is a common symptom in PD^3,4,27^. Accordingly, the late components (Components 5 and 6) were clearly and inversely associated with odor ID, a test that is partly dependent on cognitive functions^9^ such as the processing of labels and cues. Component 6 also demonstrated a direct relationship with H&Y disease severity, thus the change of the directionality of Component 6 association with H&Y disease severity compared to odor ID, indicated that PD individuals with high EBG values (i.e., good OB responses) had olfactory function more similar to Controls and were lower in the H&Y disease severity. However, when we tested the associations with a conservative 1000-randomization bootstrap test, only associations between H&Y disease severity as well as odor ID with EBG components marginally survived suggesting that these findings should be interpreted with caution and replicated in a larger sample. It is worth noting, however, that statistical permutation testing is a conservative test that controls for multiple statistical testing.

The current study is an attempt to use EBG, a measure of OB function, to aid the diagnosis of PD. However, it is not the first olfactory-related electroencephalography measure that has been used^10,11,27^. Contrary to other olfactory-related electroencephalography measures, however, the EBG method targets the OB directly, one of the first sites of insult in PD. Accordingly, the EBG measure is a promising candidate for predicting early PD development in at-risk groups. Importantly, it is also independent of cognitive performance and therefore might be a more suitable measure than behavioral odor ID tests which can be biased and affected by dementia and other cognitive impairments commonly found in PD^28,29^. Finding a reliable biomarker for PD using EEG has been the objective of many past research studies. However, a larger meta-analysis concluded that there is no evidence to support the use of electrophysiological tests as PD biomarker^30^. That said, all studies included in the meta-analysis assessed either ERPs or global power change, measures that are too general to target any specific area in the cortex. On the other hand, more recent studies assessing oscillatory responses in PD patients document more promising results where one study demonstrated that assessments of gamma power can dissociate PD from controls^31^. However, results are reported only on group level and lack sensitivity and specificity analysis. Likewise, a study employing PCA-based classification and focusing only on slow oscillations found an in- and out-sample accuracy of 85%^32^. Comparably, in the present study, we achieved a 94% accuracy, probably due to specifically targeting a region in the human brain that is a key region in early PD disease progression, the OB. That said, the EBG model’s in-sample prediction in the current dataset, despite having a higher specificity and similar sensitivity, nominally underperformed the 16-item odor ID model. One possible reason that the EBG model and the 16-item odor ID model have similar prediction error and sensitivity could be that several of the PD patients had already developed clear olfactory deficits, thereby rendering the sensitivity and prediction error of the 16-item odor ID model to be similar to the EBG model. It is worth highlighting that the two false negatives identified in the two models are different individuals. In the 16-item odor ID model, the two false negatives are both patients who did not demonstrate a reduced sense of smell, whereas these two patients were correctly assigned to the PD group in the EBG model. Hence, these differences in performance of the two models indicated that the EBG model is not merely reflecting olfactory dysfunction but relates to specific effects of PD. Using full olfactory performance testing instead of odor ID alone is likely to improve the olfactory-based methods but this improvement might not alleviate their dependency on olfactory dysfunction whereas the EBG seems to depend on PD-relevant aspects beyond mere olfactory dysfunction. The two false negatives in the EBG model did not seem to have any apparent commonalities in PD or demographic parameters that led to misclassification in the EBG model. That said, the increased specificity in the EBG model should be replicated in data with a larger sample size and more specifically, in an at-risk group. However, the general aim of this first study was to determine the feasibility of dissociating PD patients from healthy controls by means of the EBG measure. Given that the included PD patients in this proof-of-concept study had already received their PD diagnosis and the common and early pre-diagnostic occurrence of olfactory deficits, it is likely that the EBG model’s sensitivity would outperform the 16-item odor model in either pre-diagnostic PD patients, or in an at-risk group population, without clear olfactory dysfunction.

It should be stressed that these data support the use of EBG as a potential clinical tool for early PD screening but do not demonstrate an implementation-ready method. Further studies in at-risk populations and PD sub diagnoses are needed. That said, the clinical usefulness of a developed EBG-based PD screening method is considerable given that EBG assessments can be performed in a fully automatic manner with minimum dependency on the individual’s motor, cognitive, and verbal abilities, i.e., the test has the potential to be a non-invasive and easily accessible clinical test that estimates of the probability of developing PD can be based upon. However, the current method has limitations. The present electrode setup and the volume conduction do not allow us to dissociate between the left and right OB. A measure of hemispheric differences in potential early PD patients would add diagnostic value given from the clear laterality of early disease stages. However, further developments of the method might enable this in the future^33^. Moreover, patients with late stages of PD might have reduced sniff functions^34^. However, we did not find differences in the early theta band, the known sniff-dependent frequency, at the time window of relevance. Moreover, by removing the sniff-triggered event-related signal, we limited this potential difference. Also, although we use a sniff-triggered presentation, the odors are delivered at the same speed for all participants to a point high up in the nasal cavity, thus reducing potential differences in sniff magnitudes between participants^34^ rendering an odor percept largely independent of sniff magnitude. Another possible limitation of this study is the low number of included women. Relatedly, we were unable to assess potential sex-dependent effects on the results due to the small number of included women; however, it is worth noting that a recent meta-analysis indicated that the sex-dependent effect is small to non-existing in human olfaction^35^. Nonetheless, before introducing the EBG as a PD diagnostic tool, potential effects or confounds of the individual’s sex, as well as its ability to detect subtypes of PD, should be assessed in a larger sample.

To conclude, we used a non-invasive measure of OB function—the EBG—to dissociate PD from age-matched controls. The spectrogram of the EBG measures was used to assess the differences in odor processing in PDs and Controls. Specifically, we found different patterns of synchronization for PD as compared to Control and identified six specific EBG components, including gamma, beta, and theta band during both early and late time points. Using these EBG components, we marginally out-performed a clinical odor ID test while keeping a similar level of sensitivity and in-sample error prediction. Finally, these EBG components are specifically related to disease characteristics and the late components are related to olfactory tests. Taken together we argue that this method can be further developed to facilitate early diagnosis of PD and provide a robust and objective biomarker.

## Methods

### Participants

A total of 40 individuals were initially enrolled in the study. However, two individuals from the healthy control group were excluded, either due to poor EEG signal quality or identified functional anosmia. Consequently, the final sample in the study included 20 PD patients (age = 46–75, 4 women), who were clinically diagnosed with PD by a neurologist within the Karolinska University Hospital and based on the United Kingdom Parkinson’s Disease Society Brain Bank Diagnostic Criteria with Hoehn and Yahr (H&Y) severity 1–3^36^, and 18 healthy age-matched controls (age = 41–74, 4 women). PD patients were invited to the experiment by their attending physician at the end of a regular clinical visit. Also, the patients included in this study are a subsample of an ongoing longitudinal study^37^. Being the very first study on this topic and a proof-of-concept study, we included a broad set of PD patients with the only inclusion criteria that they should be in the early stages of the disease and with the exclusion criteria that they should not have been diagnosed as being anosmic prior to participation. See Table 1 for a summary of the group demographic, PD patient disease, and medication characteristics. Notably, the MDS-UPDRS and MoCA tests were collected from the patients within ±1 year from the olfactory and EEG/EBG test. All participants had no history of head trauma, were not habitual smokers, and had the intact cognitive capability. Informed signed consent was obtained from all participants and the study was approved by the Swedish Ethical Review Authority.

**Table 1:** Summary of Parkinson Disease (PD) patient and healthy control group demographics and clinical characteristics.

	PD patients	Healthy controls
*N*	20	18
Age	46–75 (mean, 61.92 ± 9.01)	41–74 (mean, 61.82 ± 8.80)
Gender	16 men, 4 women	14 men, 4 women
Disease duration	3.95 ± 2.19 (years)	–
LEDD^a^	526 ± 355	–
H&Y^b^ severity	1.65 ± 0.67	–
MoCA^c^	26.89 ± 4.46 (*n* = 18)	–
MDS-UPDRS^d^	44.23 ± 23.93 (*n* = 13)	–
UPDRS^e^	39.20 ± 17.91 (*n* = 5)	–

MoCA and MDS-/UPDRS for two patients were missing and are not included in Table 1.

^a^LEDD, Levodopa equivalent daily dose.

^b^H&Y, Hoehn & Yahr disease severity.

^c^MoCA, Montreal cognitive assessment.

^d^MDS-UPDRS, Movement disorder society unified Parkinson’s disease rating scale.

^e^Five patients had UPDRS scores available instead of MDS-UPDRS.

### Behavioral olfactory testing

Olfactory functions were determined by the clinical Sniffin’ Sticks cued odor identification test^23^ which consists of a total of 16 odors where the participant has to identify each odor given four alternatives to choose from. Normative values acquired in healthy older adults (age > 51) indicate that the normative mean score of the 16-item odor identification test is 12.62 ± 4.45^38^. Moreover, a correct ID value of 11, the 10th percentile of a score of 16–60 years old subjects, is often used as the threshold for labeling an individual as hyposmics^38^, therefore all the included healthy participants were classified as normosmic (mean, 13.78, SD ± 1.26) and scored above the threshold (i.e. 11) in the odor identification test. Of note, one individual in the Controls had the exact score of 11 who later occurred as false positive in the odor ID model. On the other hand, PD patients had an average correct score of 7.3 (SD ± 3.36) and were classified as either normosmic (*n* = 3, of whom 2 occurred as false negative in odor ID model) or hyposmic/anosmic (*n* = 17), according to the normative values.

### Odors and odor delivery method

To increase the ecological validity and the generalizability of the results three different odors were selected; grapefruit oil (Sigma Aldrich, CAS 8016-20-4), 5-nonanone (Sigma Aldrich, CAS 623-93-8), and mushroom alcohol or 1-octen-3-ol (Sigma Aldrich, CAS 3391-86-4). Odors were diluted to 11%, 4.5%, and 1.1% volume/volume concentrations, respectively, in neat diethyl phthalate (99.5% pure, Sigma Aldrich, CAS 84-66-2). The concentrations were selected based on a pilot study to evoke iso-intense olfactory perception with limited to no perceivable trigeminal perception. We picked these odors considering three parameters: (1) having low trigeminal component at the concentrations used in the study, (2) including both food and non-food odors, and (3) covering different chemical structures (we maximized the diversity of chemical structure by choosing two odor mixtures (grapefruit and mushroom) and a monomolecular symmetrical ketone [5-nonanone, a floral-like scent that was labeled and introduced to participants as “Flower”]).

All odors were delivered for a length of 1 s (condition: Odor) using a computer-controlled olfactometer with a rising time of no more than 200 ms^39^ and a total birhinal flow rate of 3 L/min. To control for the potential confounding effect on the results of laterality in PD patients, odors were delivered alternatively monorhinaly (left/right nostril) or birhinally in both PD and healthy controls. Therefore, for 2/3 of the odor trials, the odor was delivered to either left (1/3 of trials) or right nostril (1/3) with clean air with the same flow to the contralateral nostril. For the remaining 1/3 of odor trials, the odor was delivered to both nostrils in a birhinal fashion. Whether the odor was delivered to left/right or both nostrils were pseudorandomly assigned to the odor trials, controlling for a balanced division. A total of 72 odor trials per individual were tested and subsequently the power spectrums of all trials were averaged. Interspersed with the odor trials were 24 trials consisting of 1 s of 3 L/min clean air to assess neural processing during no odor nasal sniffing (condition: Sniff) as well as to assess potential tactile sensations caused by possible air fluctuation at the onset of a trial originating from valve switching. Moreover, to avoid tactile stimulation at the onset of a stimulus, a constant clean airflow of 0.3 L/min was maintained during the whole experiment, and stimuli were added to that ongoing flow. Hence, the total airflow per nostril was held constant at 1.65 L/min, a flow below the threshold known to cause nasal irritation^39^. We further prevented the potential effect of onset-expectation by using a sniff-triggered design in which all trials were initiated in phase with inhalation and unbeknown to the participant. Given that 50% of all mitral and tufted cells in the OB are locked to respiration^21^, synchronizing the onset of trials to the onset of the inhalation increase the signal-to-noise ratio (SNR). We achieved sniff triggering by monitoring the self-phased sniff pattern using a temperature pod attached next to the right nostril with an individualized threshold to trigger the olfactometer just before the nadir of the respiratory cycle and consequently match stimuli onset (factoring in the known rise-time) with nasal inspiration. The temperature change was sampled at the rate of 400 Hz (Powerlab 16/35, ADInstruments, Colorado) and processed in LabChart Pro version v7.3.8.

All timing and stimulus triggering were implemented within E-prime 2 (Psychology Software Tools, Pennsylvania). Recordings were conducted in a sound-attenuated booth with high flow-through ventilation. Participants wore headphones playing white noise throughout the experiment to mask potential auditory onset cues due to shifting airflow from the olfactometer. The volume of the noise was individually adjusted to a comfortable level. A jittered pre-stimulus interval (600–2000 ms) was implemented before the onset of each trial to further minimize the predictability of odor onset by participants. Moreover, to limit odor habituation effects, a long average inter-trial interval (14,000 ms) was used.

### Electrobulbogram measures

Four-channel EBG data were collected and digitalized at the rate of 512 Hz using active electrode EEG (ActiveTwo, BioSemi, Amsterdam, The Netherland). The EBG electrodes were placed according to Iravani and colleagues^15^ on the forehead, Fig. 1a. Prior to recording, offsets of electrodes were manually inspected within the ActiView software (BioSemi, Amsterdam, The Netherland) and those above 40 mV were adjusted until the offset reached below the threshold. The total testing session, including behavioral odor testing, required 75–85 min to complete.

EBG recordings were epoched from 2000 ms pre-stimulus to 2000 ms post-stimulus and re-referenced to the average of left and right mastoid electrodes. The signal was then notched filtered at 50 Hz to remove the power line noise and adjusted for the olfactometer trigger delay (150 ms), i.e., the time it takes from the electric triggering of the olfactometer until the odor reaches the nasal cavity. Furthermore, to remove non-signal related artifacts, trials with muscle and blink artifacts were identified by an automatic algorithm and removed from subsequent analysis. The automatic artifact identification algorithm included: filtering, Hilbert transform, z-scoring the envelope signal, and thresholding with a cutoff *z*-value of 8 for muscle and 4 for blink artifacts (for more details, please see^15^). For Controls, 72.45 ± 11.07% of Odor and 74.54 ± 12.28% of Sniff trials were identified as artifact-less and included in the analysis. Conversely, for PD, 69.24 ± 15.28% of Odor trials and 70.00 ± 15.45% Sniff trials were included in the analysis. However, given that the main concern for EBG signals is eye blink artifacts due to the close proximity of the EBG electrodes to the eyes, an additional ocular correction was performed on the remaining trials using the bilateral vertical EOG channels according to Croft and Barry^40^.

To determine odor-dependent OB responses and assess the difference between two cohorts, the pre-processed and corrected EBG signals were decomposed into time-frequency maps using multi-tapering sliding window across frequency (range: 0.5–100 Hz with step 0.5 Hz) and time (range: −2 to 2 s with step 0.01 s). Hence, the power at each time-frequency bin was estimated using 2 tapers from discrete prolate spheroidal sequences (DPSS). The length of the tapers was selected as a function of frequency such that it captures at least two cycles of each frequency bin. The frequency smoothing parameter was set to 80% of the frequency of each bin. To remove potential motor responses originating from sniffing, the baseline-corrected Odor trials were contrasted against the baseline-corrected Sniff trials for non-phase locked and phased locked responses and converted to decibels (dB). Finally, the EBG recorded responses were derived by removing the phase-locked from non-phase-locked maps and averaged over the four EBG electrodes. All the preprocessing steps and the time-frequency decompositions were carried out in the open-source toolbox Fieldtrip 2018 within MATLAB R2019b^41^.

### Statistical analysis

Initially, we evaluated the time-frequency map between PD patients and age-matched healthy controls using a nonparametric Monte Carlo permutation test. This allowed us to control for multiple testing and, because non-parametric statistics are not making assumptions about the distribution, the results from the EBG measure enabled a greater degree of generalizability. A total of 1000 permutation tests were used to determine significant clusters that were different (*p* < 0.05 and a cluster size larger than 100 bins) between PD and Control within time-frequency maps. The identified clusters were named according to their latency (e.g., Component 1, Component 2, etc. Fig. 1g). To further validate these components, we separately assessed the agreement between PD and Control individuals using intra-class correlation (ICC). The use of a non-parametric permutation test, together with assessments of the ICC, makes us believe that there is a high probability of replicating these components in an independent sample. Next, to determine if these components were associated with whether an individual belongs to the pre-defined PD or the control group, we used z-scored power values from the components as independent variables in a mixed effect logistic regression model with by-participant random intercept. Furthermore, to determine whether the EBG measure was more specific for PD than a standardized clinical odor ID test, we also dissociated PD from healthy Control using only odor ID performance as an independent variable in a similar logistic model, Fig. 2. Subsequently, the individuals who occurred as the false negatives in the EBG and odor ID model were identified, and their demographic and disease parameters were compared to pinpoint their differences, Fig. 3. We further assessed the associations between PD disease parameters and EBG response using a mixed effect linear model by examining if individual EBG components as independent variables (with by-participant random intercept) predict PD disease parameters where EBG components were found to associate with L-dopa equivalent daily dose (LEDD), H&Y disease severity, or clinical 16-item odor identification score. Finally, we performed a bootstrap test, a more conservative statistical test, thereby a 1000–resampling permutation test was carried out and in each iteration, 80% of PDs and healthy controls were randomly selected to assess the association between PD parameters, odor identification score, and EBG components, Fig. 4. All the *t*-tests were two-tailed tests where applicable.

### Reporting summary

Further information on research design is available in the Nature Research Reporting Summary linked to this article.

## Supplementary information

Supplementary Information

Reporting Summary

## Data Availability

Data is freely and publicly available at: https://osf.io/v2837/?view_only = c0a8014b29b94a22a27a7ac44fb41dba. A reporting summary for this article is available as a Supplementary Information file.

## Code availability

Code to reproduce data visualized in Figs. 1d, 1c, 2d, and 4 are available in the above-mentioned link in the Data Availability section.
