# Supplementary material for: A non-invasive olfactory bulb measure dissociates Parkinson’s patients from healthy controls and discloses disease duration
Source: NPJ Parkinsons Dis. 2021 Aug 18;7:75. doi: 10.1038/s41531-021-00220-8 (PMC8373926; doi:10.1038/s41531-021-00220-8)
Supplement: Supplementary file 1 — Supplementary Information [file 41531_2021_220_MOESM1_ESM.docx]

**A non-invasive olfactory bulb measure dissociates Parkinson’s patients from healthy controls and discloses disease duration**

Behzad Iravani, Artin Arshamian, Martin Schaefer, Per Svenningsson, & Johan N. Lundström


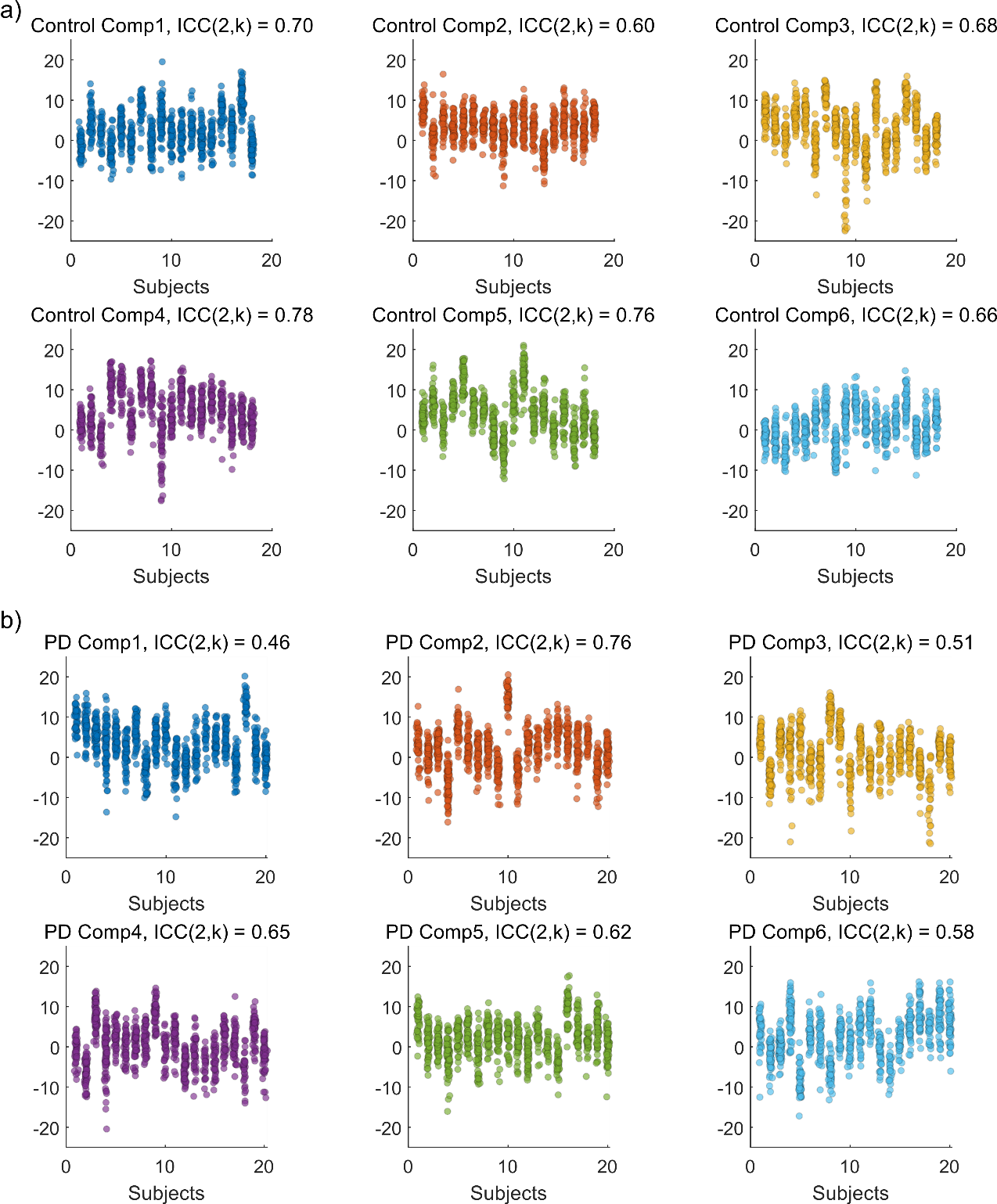


***Supplementary Figure 1. Intra-class correlation of EBG components. a****)* *The intra-class correlation (ICC) of 6 EBG components across individuals for Control group indicated moderate high agreement (.60-.70).* ***b****) Likewise, for Parkinson’s Disease patients ICC indicated medium to high agreement (.46-.76) of the EBG components. The color of the EBG components in the panel (a) and (b) corresponds with Figure 1g.*
