# Supplementary material for: A non-invasive olfactory bulb measure dissociates Parkinson’s patients from healthy controls and discloses disease duration
Source: NPJ Parkinsons Dis. 2021 Aug 18;7:75. doi: 10.1038/s41531-021-00220-8 (PMC8373926; doi:10.1038/s41531-021-00220-8)
Supplement: Supplementary file 2 — Reporting Summary [file 41531_2021_220_MOESM2_ESM.pdf]

nature research

Corresponding author(s): Behzad Iravani

Last updated by author(s): Apr 22, 2021

Reporting Summary

Nature Research wishes to improve the reproducibility of the work that we publish. This form provides structure for consistency and transparency in reporting. For further information on Nature Research policies, see our Editorial Policies and the Editorial Policy Checklist.

Statistics

For all statistical analyses, confirm that the following items are present in the figure legend, table legend, main text, or Methods section.

n/a	Confirmed	
☐	☒	The exact sample size (*n*) for each experimental group/condition, given as a discrete number and unit of measurement
☐	☒	A statement on whether measurements were taken from distinct samples or whether the same sample was measured repeatedly
☐	☒	The statistical test(s) used AND whether they are one- or two-sided<br>*Only common tests should be described solely by name; describe more complex techniques in the Methods section.*
☐	☒	A description of all covariates tested
☐	☒	A description of any assumptions or corrections, such as tests of normality and adjustment for multiple comparisons
☐	☒	A full description of the statistical parameters including central tendency (e.g. means) or other basic estimates (e.g. regression coefficient) AND variation (e.g. standard deviation) or associated estimates of uncertainty (e.g. confidence intervals)
☐	☒	For null hypothesis testing, the test statistic (e.g. *F*, *t*, *r*) with confidence intervals, effect sizes, degrees of freedom and *P* value noted<br>*Give P values as exact values whenever suitable.*
☒	☐	For Bayesian analysis, information on the choice of priors and Markov chain Monte Carlo settings
☒	☐	For hierarchical and complex designs, identification of the appropriate level for tests and full reporting of outcomes
☐	☒	Estimates of effect sizes (e.g. Cohen's *d*, Pearson's *r*), indicating how they were calculated

Our web collection on statistics for biologists contains articles on many of the points above.

Software and code

Policy information about availability of computer code

Data collection	In all experiments, E-prime 2 (Psychology Software Tools, Pennsylvania) was used to record event timing, triggering, and sniff data logging. EEG/EBG signals were sampled using active electrodes (ActiveTwo, Bio-Semi, Amsterdam, The Netherland) and online band-pass filtered at 0.01-100 Hz within the ActiView software (BioSemi, Amsterdam, The Netherland). The position of all the electrodes in stereotactic space was determined using an optical neuro-navigation system (Brain-Sight, Rogue Research, Montreal, Canada). Body micro-sway was assessed with a force plate (AccSwayPlus 591 , AMTI Massachusetts) assessing 8 axes of motion and respiration traces were digitalize using (Powerlab 16/35, ADInstruments, Colorado) and LabChart recording software (ADInstrument, Colorado).
Data analysis	All the data analysis was carried out within Matlab R2019b. Two Matlab toolboxes were used to implement the analysis including Fieldtrip 2018 for analysis EEG/EBG data and Statistical and Machine Learning toolbox for statistical analysis.

For manuscripts utilizing custom algorithms or software that are central to the research but not yet described in published literature, software must be made available to editors and reviewers. We strongly encourage code deposition in a community repository (e.g. GitHub). See the Nature Research guidelines for submitting code & software for further information.

Data

Policy information about availability of data

All manuscripts must include a data availability statement. This statement should provide the following information, where applicable:

- Accession codes, unique identifiers, or web links for publicly available datasets
- A list of figures that have associated raw data
- A description of any restrictions on data availability

Data and analysis code are freely and publicly available at https://osf.io/v2837/?view_only=c0a8014b29b94a22a27a7ac44fb41dba
Code and data to reproduce data visualized in Figures are available in the above mentioned link.

Field-specific reporting

Please select the one below that is the best fit for your research. If you are not sure, read the appropriate sections before making your selection.

☒ Life sciences ☐ Behavioural & social sciences ☐ Ecological, evolutionary & environmental sciences

For a reference copy of the document with all sections, see nature.com/documents/nr-reporting-summary-flat.pdf

Life sciences study design

All studies must disclose on these points even when the disclosure is negative.

Sample size	Given that these are the first studies of its kind, sample size was predetermined based on past experience with olfactory ERP data.
Data exclusions	Data exclusion for each study is described within each experiment. No subjects were excluded but we did trial exclusion. This is described in the method section.
Replication	We replicated the pervious finding of olfactory bulb gamma synchronization with EBG.
Randomization	None applicable.
Blinding	None applicable.

Reporting for specific materials, systems and methods

We require information from authors about some types of materials, experimental systems and methods used in many studies. Here, indicate whether each material, system or method listed is relevant to your study. If you are not sure if a list item applies to your research, read the appropriate section before selecting a response.

Materials & experimental systems

n/a	Involved in the study	
☒	☐	Antibodies
☒	☐	Eukaryotic cell lines
☒	☐	Palaeontology and archaeology
☒	☐	Animals and other organisms
☐	☒	Human research participants
☒	☐	Clinical data
☒	☐	Dual use research of concern

Methods

n/a	Involved in the study	
☒	☐	ChIP-seq
☒	☐	Flow cytometry
☒	☐	MRI-based neuroimaging

Human research participants

Policy information about studies involving human research participants

Population characteristics	The cohorts characteristics of the Parkinson patients (PD) and age-matched healthy controls are presented within Table 1. The total of 20 PD patients (4 women) with a mean age 61.92 +/- 9.01 and 18 age-matched control (4 women) with a mean age of 61.82 +/- 8.80 were tested.<br>PD patients were invited to the experiment by their attending physician at the end of a regular clinical visit. The characteristic of PD parameters are as follows: The mean levodopa equivalent daily dose was 526 +/- 355, the mean Hoehn & Yahr disease severity was 1.65 +/- 0.67, the mean Montreal cognitive assessment was 26.89 +/- 4.46 and the mean MDS-UPDRS was 44.23 +/- 23.93.
Recruitment	All patients had prior to entering the study been diagnosed with Parkinson disease by specialists at the PD clinic, Karolinska hospital, Stockholm, Sweden. Patients were invited to the experiment by our physician collaborator (Dr. Per Svenningsson).
Ethics oversight	Informed signed consent was obtained from all participants and the study was approved by the Swedish Ethical Review Authority.

Note that full information on the approval of the study protocol must also be provided in the manuscript.
